# Vibro-tactile stimulation of the neck reduces pain in people with cervical dystonia: a proof-of-concept study

**DOI:** 10.1007/s10072-024-07561-1

**Published:** 2024-05-11

**Authors:** Jiapeng Xu, Matteo Costanzo, Laura Avanzino, Davide Martino, Parisa Salehi, Stephanie Standal, Nicoletta Manzo, Parisa Alizadeh, Sara Terranova, Gaia Bonassi, Jinseok Oh, Antonella Conte, Jürgen Konczak

**Affiliations:** 1 School of Kinesiology, Human Sensorimotor Control Laboratory, University of Minnesota, Minneapolis, MN USA; 2 Department of Neuroscience, Istituto Superiore Di Sanità, Rome, Italy; 3 Department of Experimental Medicine, University of Genoa, Genoa, Italy; 4 IRCCS Policlinico San Martino, Genoa, Italy; 5 Department of Clinical Neurosciences, University of Calgary, Calgary, Canada; 6 Department of Physical Medicine & Rehabilitation, University of Minnesota, Minneapolis, MN USA; 7 IRCCS San Camillo Hospital, Venice, Italy; 8 Department of Neuroscience, University of Genoa, Genoa, Italy; 9 Present Address: Department of Human Neurosciences, Sapienza University of Rome, Rome, Italy; 10 IRCCS Neuromed, Pozzilli, IS Italy; 11 Center for Clinical Movement Science, University of Minnesota, Minneapolis, MN USA

**Keywords:** Focal dystonia, Human, Pain, Vibration, Somatosensory

## Abstract

**Background:**

Pain is a common non-motor symptom in patients with cervical dystonia (CD), severely impacting their quality of life. The pathophysiology of CD is incompletely understood but it involves altered processing of proprioceptive and pain signals.

**Objectives:**

The purpose of this proof-of-concept study was to determine if vibro-tactile stimulation (VTS)—a non-invasive form of neuromodulation targeting the somatosensory system—can modulate neck pain in people with CD.

**Methods:**

In a multi-center study, 44 CD patients received VTS to sternocleidomastoid and/or trapezius muscles for up to 45 min under 9 different stimulation conditions that either targeted a single or a pair of muscles. The primary outcome measure was a perceived pain score (PPS) rated by participants on a 100-point analogue scale.

**Results:**

During VTS, 29/44 (66%) of participants experienced a reduction in PPS of at least 10% with 17/44 (39%) reporting a reduction in pain of 50% or higher. After VTS cessation, 57% of participants still reported a 10% or higher reduction in PPS. Effects were significant at the group level and persisted for up to 20 min post-treatment. No distinct optimal stimulation profiles were identified for specific CD phenotypes. Clinical markers of disease severity or duration did not predict the degree of VTS-induced pain reduction.

**Conclusion:**

This proof-of-concept study demonstrates the potential of VTS as a new non-invasive therapeutic option for treating neck pain associated with CD. Further research needs to delineate optimal dosage and long-term effects.

**Supplementary Information:**

The online version contains supplementary material available at 10.1007/s10072-024-07561-1.

## Introduction

Cervical dystonia (CD) is a neurological disorder characterized by involuntary neck muscle contractions, determining abnormal head movements and postures, and by a wide range of non-motor aspects, including cognitive, psychiatric, or sensory disturbances [1–3]. Pain, affecting more than 70% of CD patients, significantly impacts the quality of life and may contribute to other non-motor symptoms (NMS) like sleep disturbances, anxiety, and depression, further complicating the overall management of the disorder [4–6]. Addressing pain effectively is therefore crucial to improve the overall well-being and quality of life for individuals with CD.

The main challenge in pain management in CD stems from the current lack of understanding of the underlying neural mechanisms and pathophysiology. Although pain may, in some measure, arise from overactive muscles and abnormal dystonic postures, evidence from clinical [6–9] and neurophysiological studies [10] suggests that pain in CD may have independent components not directly related to motor dysfunction. For example, maladaptive neuroplastic changes in brain regions involved in pain processing are believed to contribute to pain pathophysiology [11–13]. Furthermore, a recent neurophysiological study, aimed to test the descending inhibitory control on nociceptive neurotransmission by using a conditioned pain modulation protocol, has pointed towards a dysfunction in the endogenous inhibitory pain system [10]. Consequently, these non-motor or muscle-related components of pain might represent potential additional targets that could be explored for modulating pain in CD.

Vibro-tactile stimulation (VTS) is a non-invasive, non-pharmacological therapeutic approach that activates proprioceptive and tactile receptors embedded in the skin and muscles [14]. VTS applied to the skin can also modulate the transmission of pain signals by activating non-nociceptive nerve fibers as proposed by the gate control theory [15, 16]. Prolonged VTS, lasting over 20 seconds, has been also found to induce measurable changes in short-term cortical plasticity [17]. In addition, the application of VTS in patients affected by laryngeal dystonia can induce a significant suppression of theta band power over the left somatosensory-motor cortex and a significant rise of gamma rhythm over the right somatosensory-motor cortex, reproducing the similar neurophysiological mechanism of sensory trick [18]. Finally, recordings of single neuron activity and local field potentials from both the globus pallidum externus and internus in patients with CD revealed that neck muscle vibration modulates the firing patterns of a broad neural network, including cerebellar and cortical projections [19].

In summary, there is sufficient initial empirical evidence to indicate that cervical VTS modulates subcortical and cortical brain networks involved in the processing of somatosensory signals including pain afferents. However, at present, there are no studies that systematically evaluated if and to what extent VTS can reduce pain in those CD patients who present with pain. To close this knowledge gap, this multi-center study examined the impact of a single session of VTS on alleviating pain in patients with CD. Given the different CD phenotypes, we also aimed to determine if distinct optimal muscle stimulation profiles exist for different subtypes of CD. Finally, we investigated the potential influence of demographic and clinical characteristics in predicting the effect of VTS on CD-related pain.

## Materials and methods

### Participants

A total of 44 participants (29 female; mean age: 61.8 yrs.) with CD were recruited from 4 different collaborating centers (Minnesota: 14; Genoa: 11; Rome: 10; Calgary: 9). They were part of a larger cohort study that examined the effect of VTS on head posture. Inclusion criteria were a confirmed diagnosis of idiopathic CD without spread to other body segments and the presence of pain in the neck region. The following exclusion criteria were applied: 1) presence of other neurological disorders and 2) presence of psychiatric abnormalities affecting cognitive function that impair understanding of instructions and participation. The experimental protocol was approved by each center’s local administrative committee that provides ethical and regulatory oversight. Before enrollment, the inclusion criteria were assessed using the Toronto Western Spasmodic Torticollis Rating Scale (TWSTRS). Consent from all participants was obtained prior to testing.

### Demographic and clinical data collection

Data collection occurred during a single day and lasted approximately 3–3.5 h. Participants were screened to ensure they did not have other neurological or movement disorders besides CD. Participants receiving botulinum neurotoxin (BoNT) injection treatment were seen within two weeks before or one week after the BoNT injections to ensure that the effects of BoNT were mild and participants were symptomatic. Demographic and clinical data, such as age, gender, disease duration, and medical history were collected. In addition, information on disease duration, dosage, and frequency of BoNT injections was documented. Severity, disability and pain levels of CD participants were evaluated using the TWSTRS [20]. Its first section evaluates the amplitude of excursion of abnormal neck posture without opposing any movement while performing tasks indicated by the examiner (score: 0 to 35). The second section evaluates the degree of disability of patients and their ability to perform work, activities of daily living, or activities outside the house (score: 0 to 30). The third section evaluates the experience of pain and how it affects their everyday activities (score: 0 to 20).

### Primary outcome measure

The primary outcome measure was a perceived pain score (PPS), which participants rated on an analog scale from 0 to 100 at multiple time points with a score of 0 indicating no pain and 100 representing the most intense or maximal neck pain.

### Secondary outcome measures

Disease duration, as well as disease severity, disability and pain levels based on the TWSTRS subscale were used as predictor variables to determine VTS influence on PPS.

### Vibro-tactile stimulation device description

**Fig. 1:**
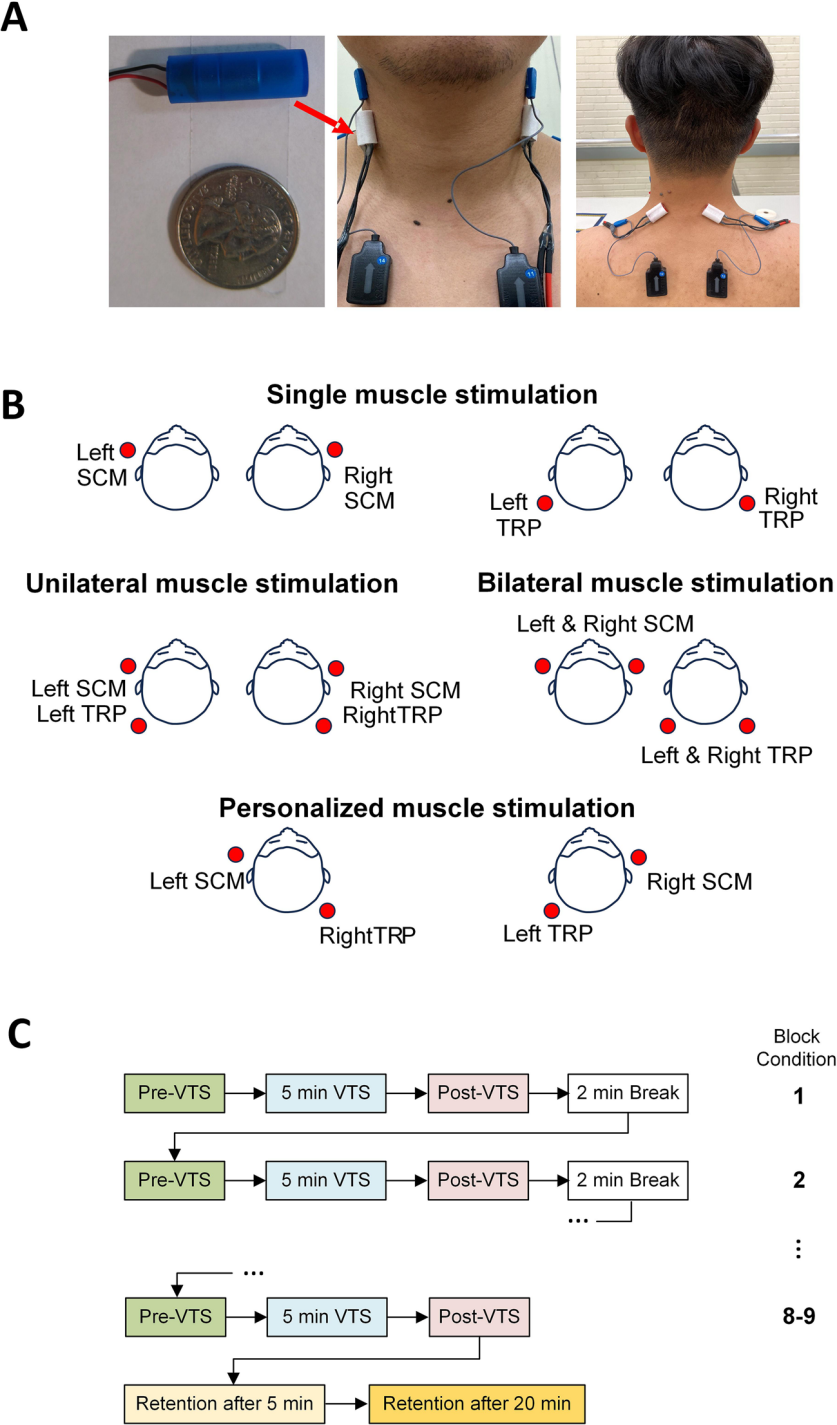
Experimental device, stimulation conditions, and experiment protocol. **A.** Image of encapsulated vibrator and vibrator placement at the sternocleidomastoid and upper trapezius muscles. **B.** Graphic of the various sites and conditions for applying vibro-tactile stimulation. **C.** Process diagram of the experimental protocol. Each block corresponds to one of the conditions illustrated in graphic B. The order of conditions was randomized across participants. *Abbreviations:* VTS, vibro-tactile stimulation; SCM, Sternocleidomastoid; TRP, Trapezius

Sternocleidomastoid (SCM) and trapezius (TRP) muscles were chosen as the targets for VTS treatment because they represent the most frequently affected muscles in CD [21]. All sites used the same experimental setup that was developed and piloted by the Minnesota center. Small electric, encapsulated vibratory motors (Precision Microdrives Ltd., London, UK, Model 307 – 100) were used to generate the vibration over the skin above the SCM and TRP muscles (see Fig. 1A). To power the motors, an AC/DC converter provided 1.2 V DC. At this voltage, the vibration frequency was approximately 100 Hz and the vibration amplitude was approximately 1.7 G. Two vibrators were taped to the target muscles to deliver vibration. Stimulation parameters were selected based on previous studies which have demonstrated that a vibration frequency of 100 Hz modulates neuronal synchronization over sensorimotor cortex in patients with laryngeal dystonia [18] and induces kinesthetic illusions in humans by acting on muscle spindle input [14, 22].

### Experimental design and procedure

The study applied a single treatment, single group experimental design. Prior to testing, a participant’s clinical status was evaluated using the TWSTRS clinical rating scale. VTS was applied to neck muscles under the following conditions: 1) single muscle stimulation (either right SCM, left SCM, right TRP, left TRP), 2) unilateral muscle stimulation (stimulating left SCM and TRP, or right SCM and TRP), 3) bilateral muscle stimulation (stimulating left and right SCM, or left and right TRP), and 4) a personalized muscle stimulation based on the treating physician’s recommendation and the participant’s BoNT injection status before the test (see Fig. 1B). During testing, participants sat comfortably in a chair. Testing started with a 2-min baseline assessment prior to attaching the vibrators to the SCM and TRP muscles, followed by the VTS treatment session with up to nine stimulation conditions. All the stimulations were delivered in a randomized order across all participants to control for order effects. Within each stimulation condition, the participant completed a 2-min pre-stimulation assessment (pre-VTS), a 5-min data collection with continuous VTS (VTS-ON), and a 2-min post-stimulation assessment (post-VTS). Between stimulation conditions, there was a 2-min break. Retention assessments were performed 5 (RET5) and 20 min (RET20) after the last VTS treatment block (see Fig. 1C). During stimulation, participants were asked to keep the trunk in an upright stationary position, and to abstain from trying to volitionally control or act against the dystonic muscle spasms. Participants rated current pain using the PPS scale at multiple time points (baseline, pre-VTS, VTS-ON, post-VTS, RET5, RET20) throughout the test.

### Statistical analysis

Statistical analysis was conducted using R (v4.2.1). Results of Shapiro–Wilk test indicated that the PPS data did not follow a normal distribution (W = 0.91, *p* < 0.05). Consequently, non-parametric statistics were applied for analysis. Wilcoxon Signed-Rank tests were performed to compare the PPS obtained at the pre-VTS of the first treatment block (initial pre-VTS), the post-VTS of the final treatment block (final post-VTS) and the retention time points (RET5 and RET20, see Fig. 1C). The initial significance level was set to *p* = 0.05. The Benjamini–Hochberg method was used to correct for multiple comparisons where appropriate.

To determine the optimal muscle stimulation profile for each CD manifestation, the CD participants were divided into six subgroups according to their CD manifestation: left/right torticollis, left/right laterocollis, anterocollis, and retrocollis. Participants presenting with complex dystonic patterns were assigned to more than one group. For each participant, all stimulation conditions that resulted in more than 10% of relative improvement in PPS were identified. The mean relative improvement across participants and the response rate were summarized for each stimulation condition within each CD manifestation group based on PPS. The responder rate of a particular stimulation condition was defined as the ratio between the number of participants who showed 10% or higher improvement in PPS and the number of participants within a CD subgroup. The optimal muscle stimulation profile for each CD manifestation was identified by selecting the stimulation condition that resulted in the largest mean relative improvement or the highest response rate for each of the six CD subgroups. When selecting the optimal muscle stimulation profile based on the mean relative improvement metric, data from fewer than three individuals per condition were not considered for this analysis.

A stepwise model selection method (stepAIC function, MASS package in R, version 7.3–58.1) was performed to determine the optimal linear regression model that predicts the relative change in PPS [23]. The relative change in PPS at VTS-ON was the dependent variable and demographic and clinical variables were independent variable candidates. Model with the lowest Akaike's Information Criterion was selected as the optimal model [24]. Details of all independent variable candidates can be found in Table 1 in Supplementary Materials.

## Results

### Demographic and clinical characteristics of the sample

Mean disease duration of the participant sample was 13.6 ± SD 12.8 years. Using the score of 2nd subscale section of TWSTRS-2 as a measure of motor symptoms severity, yielded a mean severity score of 16 ± SD 6.9, while the mean subscale scores for disability and pain were 11.2 ± SD 6.1 and 8 ± SD 5.5, respectively. Thirty-nine out of forty-four participants were treated with BoNT, with a mean interval of 12.7 ± SD 2.7 weeks between injection sessions. With respect to clinical phenotype, 41/44 (89%) of patients presented with torticollis, laterocollis was present in 35/44 (79.5%), anterocollis in 12/44 (27%) and retrocollis in 11/44 (25%). A total of 39 (89%) out of 44 participants presented with a complex symptom pattern of CD (i.e., presenting with two or more phenotypes).

### Effect of VTS on pain in CD

To differentiate between responders and non-responders to VTS, we applied a 10% or higher relative reduction in PPS during the application of VTS for at least one stimulation condition as the threshold. Accordingly, 66% (29/44) of the study cohort were classified as responders and 44% (15/44) as non-responders. Among the 29 responders, 20 participants responded to VTS during one to three stimulation conditions, while 9 participants responded to more than three stimulation conditions. The use of an effective sensory trick did not clearly differentiate between responders and non-responders. A total of 82% (23/28; *n* = 1 missing information) of responders and 60% (9/15) of non-responders reported the use of an effective sensory trick.

**Fig. 2:**
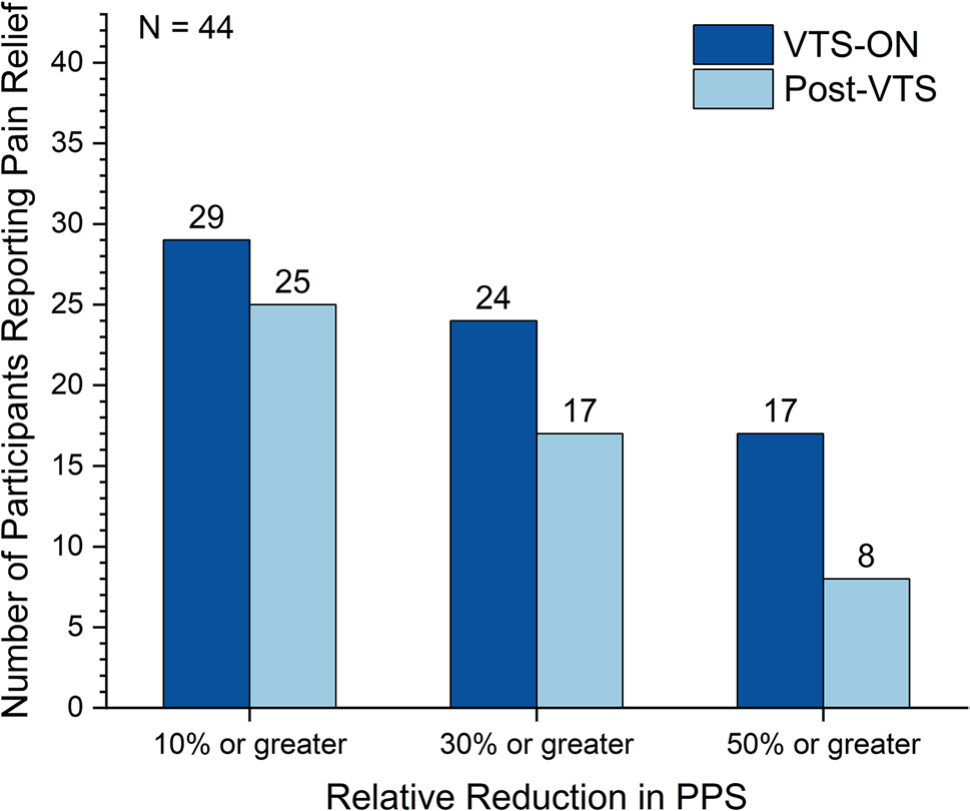
Magnitude and frequency of pain relief due to vibro-tactile stimulation (VTS). Shown is the relative change in perceived pain score (PPS) during VTS-ON and post-VTS relative to the pre-VTS baseline for each participant’s most effective stimulation condition

For each participant, the stimulation condition yielding the largest relative reduction in PPS was determined as the most effective stimulation. Analysis of the relative reduction in PPS showed that 66% (29/44) of participants reported a 10% or higher relative improvement in PPS, with 39% (17/44) achieving a 50% or higher relative improvement in PPS during the application of VTS for a participant’s most effective stimulation condition. After cessation of VTS under the most effective stimulation condition, 57% (25/44) of participants still showed 10% or higher relative improvement in PPS (see Fig. 2).

### Retention of therapeutic effects after cessation of VTS

**Fig. 3:**
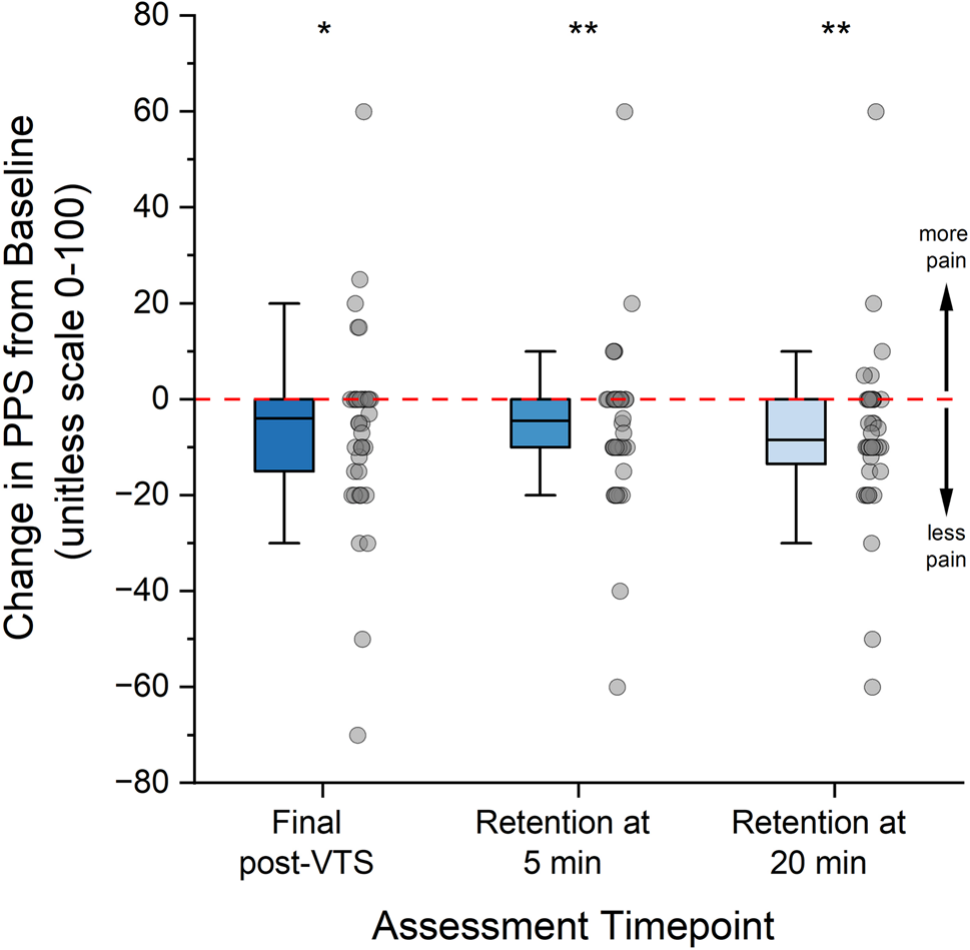
Change in pain score due to cervical vibro-tactile stimulation (VTS). Each data point (grey circle) represents the change in perceived pain score (PPS) relative to baseline (pre-VTS of the first VTS treatment block) for an individual participant. PPS was assessed at three time points: Final post-VTS = immediately after the entire VTS treatment session, Retention at 5 and 20 min = assessment at 5 and 20 min after the cessation of VTS. Negative values indicate a relief of pain. The lower and upper whiskers represent the 5th and 95th percentile. Solid lines in each box indicates the median. * indicates a *p*-value < 0.05, ** indicates a *p*-value < 0.01 for the comparisons relative to the pre-VTS of the first block

PPS obtained before the entire VTS session (initial pre-VTS), immediately after VTS (final post-VTS), and at 5 and 20 min after cessation of VTS (RET5, and at RET20) were analyzed. Significantly smaller pain scores were observed at multiple time points when compared to the initial pre-VTS (final post-VTS: Z = -2.43, p_adj_ = 0.02; RET5: Z = -2.81, p_adj_ < 0.01; RET20: Z = -3.53, p_adj_ < 0.01). The median paired difference of PPS was -4 (Q1, Q3: -15, 0) between the final post-VTS and the initial pre-VTS, was -4.5 (Q1, Q3: -10, 0) between the RET5 and the initial pre-VTS, and was -8.5 (Q1, Q3: -12.75, 0) between the RET20 and the initial pre-VTS (see Fig. 3).

### Identification of the optimal muscle stimulation profile for each CD manifestation

**Fig. 4:**
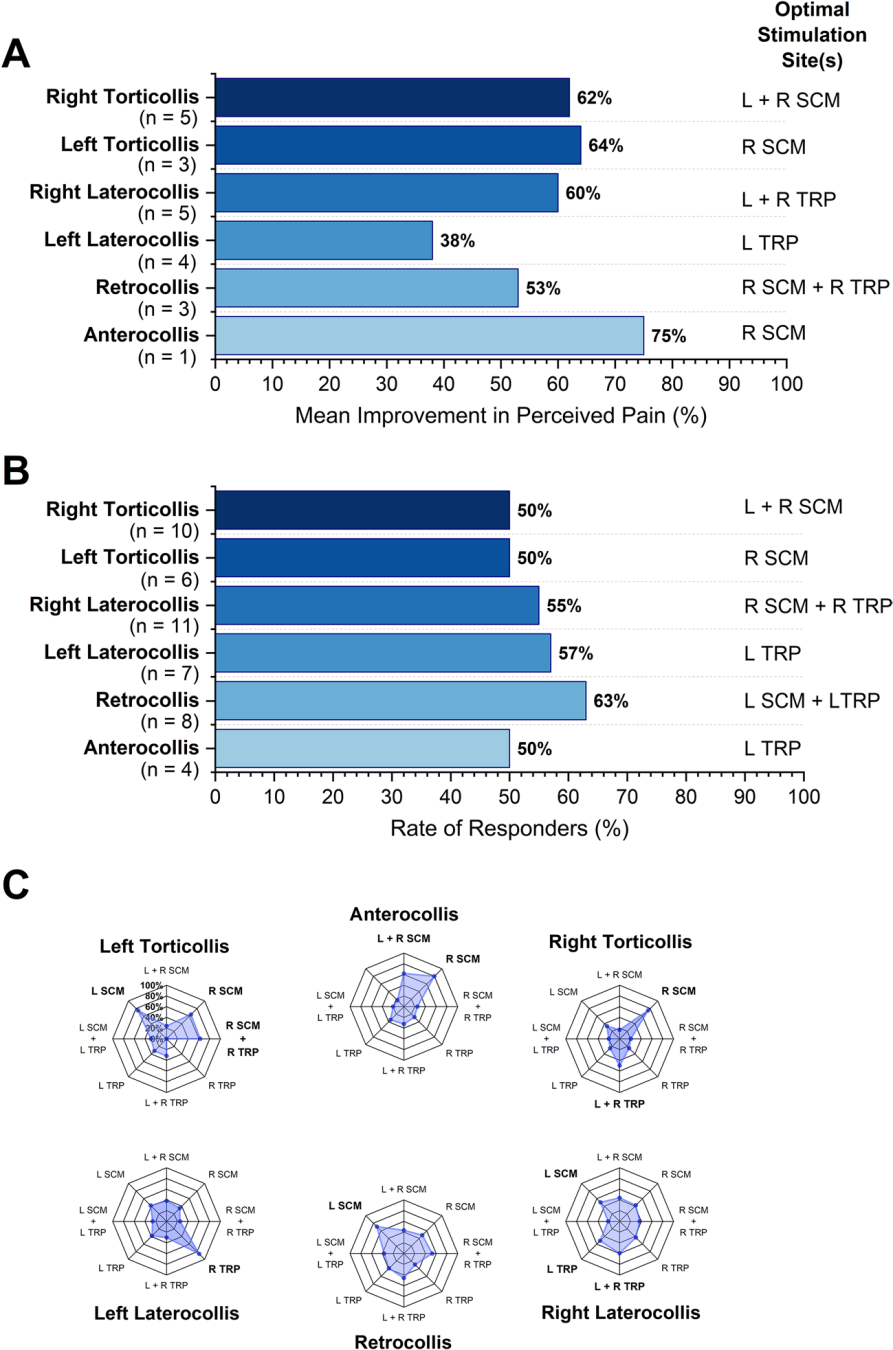
VTS-induced reduction in pain and response rate by CD manifestation. **A.** Shown is the mean relative improvement across participants for the optimal muscle stimulation condition for each of the six CD manifestations. Next to each bar, the related optimal stimulation site/condition yielding the largest mean relative pain reduction is indicated. Note that for anterocollis, none of the stimulation conditions had more than two participants who showed 10% or higher relative improvement in PPS. **B.** Shown is the optimal muscle stimulation condition yielding the highest response rate for each CD manifestation subgroup. **C.** Shown is the mean relative improvement across participants for each stimulation condition for each of the six CD manifestation subgroup. Note that for some stimulation conditions, the mean relative improvement was calculated with data from one or two participants and these conditions were not considered when selecting optimal muscle stimulation condition within that CD manifestation subgroup Abbreviations VTS, Vibro-tactile stimulation SCM, Sternocleidomastoid TRP, Trapezius

For each CD phenotype, we determined the stimulation conditions that induced the largest mean relative improvement in PPS (see Fig. 4A). In addition, the stimulation condition with the highest PPS-based responder rate was identified for each CD manifestation (see Fig. 4B). The personalized stimulation condition that focused on the dystonic muscles receiving BoNT induced a 20–42% mean relative improvement in PPS, which was not necessarily the condition that reduced perceived pain the most. The radar plot Fig. 4C summarizes the mean relative improvement in PPS for each stimulation condition for each of the six CD subtypes. As can be seen, specific stimulation sites or conditions yielded higher reduction in PPS than others. For example, for right and left torticollis VTS to single or bilateral SCM muscles was most beneficial while trapezius muscle stimulation resulted in lower reductions at the group level. In contrast, for left torticollis right TRP stimulation yielded the largest relative reduction in PPS.

### Demographic and clinical characteristics as predictors of VTS effects on CD-related pain

The adjusted linear regression model showed that the relative change in PPS was associated with disease duration, disability scores, retrocollis subtype and CD severity. On average, a one-year increase in disease duration was associated with 1% (95% CI: 0 ~ 2%, *p* = 0.05) greater relative improvement in PPS after controlling for the other variables. One-point increase in the TWSTRS disability subscale was associated with 3% (95% CI: 0 ~ 5%, *p* = 0.03) greater relative improvement in PPS after controlling for the other variables. Participants who have retrocollis showed 22% (95% CI: -3 ~ 48%, *p* = 0.09) greater relative improvement in PPS than participants who do not have such manifestation after controlling for the other variables. One-point increase of the TWSTRS severity subscale was associated with 3% (95% CI: 1 ~ 5%, *p* < 0.01) less relative improvement in PPS after controlling for the other variables. However, the entire model explained only 17% of variation in the relative change in PPS between pre-VTS vs VTS-ON under the most effective stimulation (R^2^_adj_ = 0.17) indicating that factors of clinical presentation, disease severity and duration were not significant predictors of experienced pain reduction.

## Discussion

This proof-of-concept study is the first investigation to systematically evaluate the effect of cervical VTS on pain in a cohort of CD patients. Results indicate that the majority of participants perceived pain relief during the application of VTS with 66% of them indicating a 10% or higher relative improvement in one or more VTS stimulation conditions, and 39% of participants reporting a relative pain reduction of 50% or higher. Pain relief was retained for up to 20 min following the cessation of VTS. The various CD phenotypes did not show distinct optimal stimulation profile and the stimulation site yielding the largest pain reduction did not necessarily correspond to the muscles that were targets of BoNT injection. Finally, disease duration, disease severity and CD phenotype did not meaningfully predict the extent of experienced pain reduction.

### Possible neurophysiological and neuromechanical mechanisms underlying the observed pain relief

At present, the pathophysiology of CD-related pain is still incompletely understood. However, there is evidence that pain associated with CD is not entirely explained by abnormal muscle contractions, because BoNT treatment may improve dystonic contractions without relieving pain and the severity of motor signs of CD does not always correlate with the duration or intensity of neck pain [9]. Although this study was not designed to elucidate the neurophysiological mechanism behind the effectiveness of VTS in reducing pain, it is imperative to consider plausible scenarios of how a somatosensory signal due to VTS alters or modulates the processing of somatosensory nociceptive afferents.

A first scenario to entertain is that VTS simply constitutes a form of mechanical muscle massage that induces temporary relief. Yet, this mechanism unlikely explains the observed effects because the vibrators in this study were small and not designed to provide sufficient amplitude to mechanically massage large sections of the neck and to penetrate deeper muscle layers. The type of vibration applied could only reach a limited area of superficial muscle fibers, which makes the assumption of a wide-spread neck muscle massage effect not plausible. However, there are studies showing that mechanical vibration can induce pain relief [16, 25]. For example, in a cohort of 366 patients experiencing acute or chronic musculoskeletal pain, the application of light pressure vibration at a frequency of 100 Hz, which is identical to the stimulation frequency used in our study, effectively provided temporary pain relief for 69% of the participants [16].

A second scenario centers on the hypothesis that the observed pain relief during and after VTS constitutes a placebo effect attributable to psychological mechanisms of expectation. It could be argued that the failure to find a single optimal muscle stimulation site for each CD phenotype on a group level supports the notion that VTS induces an unspecific placebo effect. However, one needs consider the known pathophysiology of CD that is characterized by abnormal overlapping neural representations of body and muscle systems in somatosensory and motor cortex. Such “smeared” cortical representations could also explain why responses to VTS were not specific to a single muscle site [26]. Moreover, for individual participants reporting pain relief, the perceived reduction in pain occurred quickly within seconds and was not identical across all muscle sites, features that are difficult to reconcile with an unspecific placebo response. Relatedly, the group data in Fig. 4C underline the notion that certain muscle sites induced higher benefits than others for specific phenotypes. In addition, a case report found that VTS applied to the trapezius muscle was effective in restoring an upright head posture in a CD patient [27]. In contrast, sham conditions such as transcutaneous electrical nerve stimulation were ineffective. In conclusion, while one cannot exclude the possibility that contextual effects contributed to perceived pain relief, the observed responses to VTS are not fully explained by the assumption of an unspecific placebo effect.

One possible mechanism behind the effectiveness of VTS for improving pain in CD involves the modulation of nociceptive sensory signals within the dorsal horn of the spinal cord by faster A-beta fibers responsible for transmitting vibrotactile sensory inputs as postulated by the gate control theory of Melzack and Wall [15]. However, one needs to consider that the therapeutic effects of VTS on pain relief persisted after the cessation of VTS, suggesting that its neural effect may go beyond the competitive inhibition of noxious stimuli at the spinal cord level. An alternative, not mutually exclusive scenario is that VTS modulates a complex network of supraspinal structures involved in pain processing such as the nucleus reticularis dorsalis and its connections to the striatum, thalamus, the dorsolateral prefrontal cortex and the midcingulate cortex [28–30]. For example, recent work confirmed that neck muscle vibration of patients with CD notably increased the regularity of neck-sensitive neurons in globus pallidus internus [19]. Moreover, it has been documented that VTS normalizes the excessive synchronization of neurons in somatosensory and motor cortex in people with laryngeal dystonia – a response that mirrors the electrocortical response associated with an effective sensory trick in CD [18, 31]. In summary, there is converging neurophysiological evidence that neck muscle VTS induces fast, repeatable responses in a wide neural network comprising subcortical and cortical structures involved in somatosensory signal processing.

### Study control and limitations

We took precautions to exclude methodological biases and to ensure data validity. To minimize the risk of misdiagnosis, all study participants underwent evaluation by specialists in movement disorders. To avoid any confounding effect of prior BoNT injections, we scheduled the experimental session within a fixed timeframe, either two weeks before or one week after a new round of injections. All investigators received standardized training, including detailed instructions on how to apply cervical VTS treatment and how to follow the protocol developed by the coordinating center (Minnesota). Regular quality control checks were conducted by personnel of the coordinating center to ensure adherence to the standardized protocol at the satellite centers (Calgary, Genoa, Rome). Finally, the type of vibratory motors to apply VTS and their settings were identical across sites to ensure that compatibility across sites.

We recognize several limitations: First, the current study only assessed acute effects to VTS in a single instance. We have no systematic knowledge of possible longitudinal VTS effects. Second, VTS was applied to the SCM and TRP muscles, which are the two most commonly affected muscles in CD [21]. Other cervical muscles such as splenius capitis, scalenus, or platysma, which can also be affected by CD were not stimulated in this study. Third, we employed a single frequency and intensity for the VTS treatment. Investigating the effects of varying frequencies and intensities that target different mechanoreceptors is imperative to better understand the neurophysiology underlying VTS. Finally, future clinical trials should incorporate sham conditions to validate these initial findings and to discern the true efficacy of VTS. Moreover, a systematic follow-up is needed to obtain knowledge on what muscle(s) to target for a specific CD phenotype and/or localization of pain. The present data show that for a given CD phenotype several stimulation sites can lead to pain reduction and both single and dual muscle stimulation may be effective (see Fig. 4C). In addition, the current data indicate that that for some phenotypes a single stimulation site is most effective (e.g., right TRP for left laterocollis), yet for other phenotypes such specificity was not found. At present, we have an incomplete understanding why this is the case.

## Conclusions

The study data provide proof-of-concept that cervical VTS can induce fast-acting pain relief in CD that persists for up to 20 min after the cessation of VTS. Further systematic investigations are necessary to determine long-term effects, optimal dosage and to develop evidence-based treatment strategies for CD patients. To establish VTS as a new therapeutic option for treating neck pain associated with CD, additional research is needed that compares the effectiveness of VTS to BoNT injections in alleviating CD-related pain and evaluates potential synergistic effects of combining VTS with BoTN injections.

## Supplementary Information

Below is the link to the electronic supplementary material.

Supplementary file1 (DOCX 15 KB)

Supplementary file2 (DOCX 29 KB)

## Data Availability

Data of the study are available upon reasonable request from the senior author.
