# Supplementary material for: Vibro-tactile stimulation of the neck reduces pain in people with cervical dystonia: a proof-of-concept study
Source: Neurol Sci. 2024 May 11;45(10):4847–56. doi: 10.1007/s10072-024-07561-1 (PMC11422418; doi:10.1007/s10072-024-07561-1)
Supplement: Supplementary file 1 — Supplementary file1 (DOCX 15 KB) [file 10072_2024_7561_MOESM1_ESM.docx]

**Supplementary Materials**

**Table 1.** The name, possible value and description of the independent variable candidates that were included for stepwise model selection

| Variable Name | Value | Description |
| --- | --- | --- |
| Sex | 0, 1 | Level 0 indicates female. Level 1 indicates male. |
| Age | 21~82 | (Years) Participant’s age when they attended the testing. |
| CD Duration | 0.3~50 | (Years) Duration of CD after diagnosis. |
| Severity | 3~35 | Severity subscale of TWSTRS. |
| Disability | 0~25 | Disability subscale of TWSTRS. |
| Pain | 0~21.5 | Pain subscale of TWSTRS. |
| Sensory Trick | 0, 1, 2 | Effectiveness of sensory trick. Level 0 indicates majority or all the CD symptoms would be relieved by performing sensory trick. Level 1 indicates partial CD symptoms would be relieved by performing sensory trick. Level 2 means the sensory trick does not work. |
| Torticollis | 0, 1 | Level 1 indicates the participant has torticollis. |
| Laterocollis | 0, 1 | Level 1 indicates the participant has laterocollis. |
| Anterocollis | 0, 1 | Level 1 indicates the participant has anterocollis. |
| Retrocollis | 0, 1 | Level 1 indicates the participant has retrocillis. |
| Tremor | 0, 1 | Level 1 indicates the participant have a tremor. |
| BoNT Responder | 0, 1 | Level 1 indicates participant benefit from BoNT injection. |
| BoNT History | 0~50 | (Years) Duration of BoNT injection. |
| BoNT Bofore Test | 0, 1 | Level 1 indicates participant did not receive BoNT injection before the testing. |
| Left SCM BoNT | 0, 1 | Level 1 indicates left SCM received BoNT before the testing. |
| Right SCM BoNT | 0, 1 | Level 1 indicates right SCM received BoNT before the testing. |
| Left TRP BoNT | 0, 1 | Level 1 indicates left TRP received BoNT beofore the testing. |
| Right TRP BoNT | 0, 1 | Level 1 indicates right TRP received BoNT beofore the testing. |

***Abbreviations:*** TWTSRS, revised Toronto Western Spasmodic Torticollis Rating Scales; BoNT, Botulinum Toxin; SCM, sternocleidomastoid muscle; TRP, trapezius muscle
