# Supplementary material for: Vibro-tactile stimulation of the neck reduces pain in people with cervical dystonia: a proof-of-concept study
Source: Neurol Sci. 2024 May 11;45(10):4847–56. doi: 10.1007/s10072-024-07561-1 (PMC11422418; doi:10.1007/s10072-024-07561-1)
Supplement: Supplementary file 2 — Supplementary file2 (DOCX 29 KB) [file 10072_2024_7561_MOESM2_ESM.docx]

**Supplementary Materials**

**Table 2.** Demographic and clinical information of participants. The severity, disability, and pain columns refer to the subscales from TWSTRS. Initial PPS = perceived pain score prior to VTS application.

| Subject  ID | Sex | Age (years) | Severity  (0 – 35) | Disability (0 – 30) | Pain  (0 – 20) | Initial PPS  (0 - 100) | CD Manifestations |
| --- | --- | --- | --- | --- | --- | --- | --- |
| RO003 | F | 48 | 13 | 10 | 2 | 40 | Right torticollis |
| RO004 | F | 73 | 11 | 5 | 6 | 30 | Right torticollis, left laterocollis, retrocollis |
| RO005 | F | 61 | 15 | 10 | 8 | 40 | Right torticollis, left laterocollis |
| RO006 | F | 66 | 9 | 11 | 3 | 20 | Left torticollis, right laterocollis |
| RO007 | F | 81 | 6 | 0 | 3 | 40 | Right laterocollis, retrocollis |
| RO008 | F | 78 | 6 | 0 | 2 | 10 | Right torticollis, left laterocollis |
| RO010 | M | 45 | 9 | 7 | 2 | 20 | Left torticollis, left laterocollis |
| RO011 | M | 41 | 23 | 16 | 6 | 10 | Right torticollis, left laterocollis, retrocollis |
| RO012 | F | 54 | 10 | 9 | 4 | 40 | Right torticollis, anterocollis |
| RO014 | F | 61 | 8 | 0 | 3 | 30 | Left torticollis, anterocollis |
| RO015 | F | 51 | 13 | 14 | 18 | 80 | Right laterocollis |
| GE002 | M | 58 | 17 | 1 | 1.25 | 20 | Left torticollis, left laterocollis |
| GE004 | M | 52 | 19 | 11 | 11.25 | 30 | Right torticollis, right laterocollis |
| GE008 | F | 67 | 15 | 9 | 7.5 | 40 | Right torticollis, retrocollis |
| GE009 | F | 62 | 20 | 14 | 6.25 | 20 | Left torticollis, left laterocollis |
| GE012 | M | 76 | 18 | 18 | 12.25 | 100 | Left torticollis, anterocollis |
| GE013 | F | 72 | 29 | 25 | 17 | 100 | Left torticollis, left laterocollis, anterocollis |
| GE014 | M | 55 | 17 | 6 | 4 | 10 | Right torticollis |
| GE015 | M | 82 | 31 | 18 | 0 | 10 | Right torticollis, right laterocollis, anterocollis |
| GE017 | M | 48 | 18 | 15 | 5.5 | 20 | Right torticollis |
| GE018 | M | 70 | 35 | 10 | 21.5 | 10 | Right torticollis, right laterocollis, anterocollis |
| MN004 | F | 60 | 6 | 10 | 12.75 | 20 | Right torticollis, left laterocollis, retrocollis |
| MN006 | F | 66 | 22 | 7 | 7.25 | 35 | Left torticollis, left laterocollis, retrocollis |
| MN007 | F | 74 | 20 | 13 | 13 | 30 | Left torticollis, right laterocollis, anterocollis |
| MN009 | M | 51 | 15 | 18 | 13.25 | 20 | Left torticollis, left laterocollis |
| MN010 | F | 64 | 20 | 10 | 16 | 60 | Right torticollis, right laterocollis, anterocollis |
| MN011 | F | 43 | 14 | 12 | 10.75 | 20 | Right torticollis, right laterocollis, anterocollis |
| MN012 | F | 63 | 17 | 11 | 6 | 8 | Left torticollis, left laterocollis, retrocollis |
| MN015 | M | 62 | 22 | 23 | 13 | 60 | Right torticollis, retrocollis |
| MN016 | F | 57 | 21 | 19 | 15.25 | 70 | Right torticollis, right laterocollis, anterocollis |
| MN017 | F | 68 | 18 | 9 | 9.25 | 20 | Left torticollis, left laterocollis, retrocollis |
| MN018 | F | 53 | 3 | 1 | 7.75 | 30 | Right torticollis, left laterocollis |
| MN019 | M | 54 | 8 | 7 | 4 | 15 | Right torticollis, retrocollis |
| MN020 | M | 68 | 28 | 6 | 5.75 | 20 | Right torticollis, left laterocollis, retrocollis |
| MN021 | F | 60 | 15 | 17 | 18 | 90 | Left torticollis, left laterocollis |
| CAL001 | F | 50 | 14 | 11 | 11 | 65 | Left torticollis, left laterocollis, anterocollis |
| CAL002 | F | 65 | 15 | 12 | 11 | 30 | Left torticollis, right laterocollis |
| CAL004 | F | 64 | 21 | 12 | 11 | 40 | Right torticollis, right laterocollis |
| CAL009 | F | 73 | 12 | 20 | 0 | 20 | Left Torticollis, right laterocollis |
| CAL011 | M | 68 | 14 | 13 | 0 | 20 | Right torticollis, left laterocollis |
| CAL012 | F | 64 | Missing | Missing | Missing | 40 | Right laterocollis |
| CAL015 | F | 64 | 18 | 19 | 8 | 80 | Right torticollis, left laterocollis |
| CAL016 | F | 60 | 10 | 8 | 2 | 30 | Right torticollis, right laterocollis, anterocollis |
| CAL019 | M | 68 | 13 | 16 | 7 | 10 | Right torticollis, right laterocollis |
